# High Levels of Fine Particulate Matter (PM_2.5_) Concentrations from Burning Solid Fuels in Rural Households of Butajira, Ethiopia

**DOI:** 10.3390/ijerph18136942

**Published:** 2021-06-29

**Authors:** Mulugeta Tamire, Abera Kumie, Adamu Addissie, Mulugeta Ayalew, Johan Boman, Susann Skovbjerg, Rune Andersson, Mona Lärstad

**Affiliations:** 1 Department of Preventive Medicine, School of Public Health, Addis Ababa University, Addis Ababa P.O. Box 9086, Ethiopia; aberakumie2@yahoo.com (A.K.); adamuaddissie@gmail.com (A.A.); mulugetaayalewju@gmail.com (M.A.); 2 Department of Occupational and Environmental Medicine, Institute of Medicine, Sahlgrenska Academy, University of Gothenburg, 41390 Gothenburg, Sweden; mona.larstad@vgregion.se; 3 Department of Chemistry and Molecular Biology, Atmospheric Science, University of Gothenburg, 41696 Gothenburg, Sweden; johan.boman@gu.se; 4 Department of Infectious Diseases, Institute of Biomedicine, Sahlgrenska Academy, University of Gothenburg, 41345 Gothenburg, Sweden; susann.skovbjerg@vgregion.se (S.S.); rune.andersson@gu.se (R.A.); 5 Department of Clinical Microbiology, Sahlgrenska University Hospital, Region Västra Götaland, 41346 Gothenburg, Sweden; 6 Department of Respiratory Medicine and Allergology, Institute of Medicine, Sahlgrenska University Hospital, 41390 Gothenburg, Sweden

**Keywords:** solid fuel use, household air pollution, particulate matter (PM_2.5_), Ethiopia

## Abstract

The use of solid fuel, known to emit pollutants which cause damage to human health, is the primary energy option in Ethiopia. Thus, the aim of this study was to measure the level of household air pollution by using the 24-h mean concentration of fine particulate matter (PM_2.5_) in 150 randomly recruited households in rural Butajira, Ethiopia. Data relating to household and cooking practices were obtained by conducting face-to-face interviews with the mothers. The 24-h mean (standard deviation) and median PM_2.5_ concentrations were 410 (220) and 340 µg/m^3^, respectively. Households using only traditional stoves and those who did not open the door or a window during cooking had a significantly higher mean concentration compared with their counterparts. There is a statistically significant correlation between the mean concentration of PM_2.5_ and the self-reported cooking duration. The pollution level was up to 16 times higher than the WHO 24-h guideline limit of 25 μg/m^3,^ thus leaving the mothers and children who spend the most time at the domestic hearth at risk of the adverse health effects from solid fuel use in Ethiopia. Thus, effective short- and long-term interventions are urgently needed.

## 1. Introduction

The burning of wood and other solid fuels such as crop residues, charcoal and dung for cooking are major sources of particulate air pollution [1]. The resulting combustion particles are largely in the fine particulate matter (PM_2.5_) mode, i.e., with an aerodynamic diameter less than 2.5 micrometers (µm) [1,2]. The cooking practice on open fires and inefficient stoves produces high pollutant emissions which account for a large proportion of damage to human health especially in low- and middle-income countries. The most vulnerable to these high exposure levels are women and young children as they spend most of their time near the domestic hearth [1,3,4,5,6].

Exposure to household air pollution in adults has been associated with various diseases such as chronic obstructive pulmonary disease (COPD), asthma, cancers of the lungs and nasopharynx, stroke, ischemic heart diseases and diseases of the eye [1,3,7,8,9,10]. It is also associated with low birth weight and, of particular concern, acute lower respiratory infections such as pneumonia among children less than five years of age [1,4,11,12]. In total, nearly four million premature deaths per year from illnesses attributable to household air pollution are reported globally [1].

In order to halt the vast range of public health risks associated with household air pollution, the World Health Organization (WHO) guideline stipulates a PM_2.5_ concentration below 10 µg/m^3^ annual mean and 25 µg/m^3^ 24-h mean for maintaining safe indoor air quality in low- and middle-income countries [13]. High indoor PM_2.5_ concentration is still a problem in various parts of the world [14], far exceeding the given limits in countries where a large proportion of the population depends on solid fuel use for cooking and heating [15]. Together with the population growth in the Sub-Saharan Africa region [16], the problem of household air pollution and related health problems, including the negative association of this with life expectancy in the long term [17], puts Sub-Saharan Africa singularly at a higher risk, when compared with other parts of the world [12,18].

In Ethiopia, the use of biomass fuel, mainly wood, is the only energy option in nearly all rural parts of the country. Our previous study showed that respiratory symptoms were twice as common among mothers in rural Butajira, where biomass fuel is almost the sole source of energy, when compared with those mothers in Addis Ababa, who were using cleaner energy sources [19]. Other studies in the country also reported solid fuel use to be associated with low birth weight [20], acute respiratory infection in younger children [21,22] and poor asthma control in clinical patients [23].

Gathering evidence on the level of household air pollution in a country with a population of over 100 million (80% rural) is vital to estimate the public health impact of this on respiratory health. The aim was thus to measure the 24-h mean concentration of household PM_2.5_ in one rural area of Ethiopia. The findings highlight the burden of household air pollution for policy-makers but could also be used as a baseline for future interventions.

## 2. Methods and Materials

### 2.1. Study Design and Period

A cross-sectional study was conducted during two periods: from August to October 2018 and from January to March 2019. The former period includes a rainy season, while the latter months are the dry season in the area, thus allowing comparison between different seasons.

### 2.2. Study Area and Recruitment of Households

This study was conducted in rural Butajira, at five kebeles (the lowest administrative unit), namely Dirama, Dobena, Misrak Meskan, Shersherabido and Wurib in the Gurage zone of the Southern Nations and Nationalities and Peoples Region (SNNPR), which is situated approximately 136 km south of Addis Ababa at a moderate altitude of 2131 m. The selected rural kebeles were all included in the Demographic Surveillance System (DSS) of Addis Ababa University Rural Health Programme [24] considering their proximity to the district city, Butajira from all directions. Overall, 150 households were included. These were randomly selected from the five villages which were denoted by unique codes assigned to them by the DSS office. The recruitment of households from Wurib and Dobena villages was slightly higher based of the total number of households in these villages.

### 2.3. Interview Based on Questionnaires

The data collection expert, who has a Masters of Public Health in Environmental and Occupational Health was trained in the use of the measuring instruments and the filling out of the questionnaires. He approached the households with guidance from data enumerators from Addis Ababa University who were residents of the same villages. Face-to-face interviews were conducted both before and after the PM_2.5_ measurements; the questionnaires comprised questions relating to family size, housing characteristics, types of stove, fuel use and cooking processes, such as cooking duration and frequency. The questionnaires also included questions about the weather during the monitoring period and ventilation activities. None of the household members was a current smoker and all used chargeable batteries for lighting at night. Thus, there were no additional sources of household air pollution other than the burning process.

### 2.4. Quantification and Monitoring of PM_2.5_

In our study, the Particle and Temperature Sensor (PATS+) instrument was used along with the Platform for Integrated Cook stove Assessment (PICA) software from Berkeley Air Monitoring Group [25] to monitor the concentration of PM_2.5_. PATS+ is a portable, data-logging, battery-operated instrument measuring real-time particulate matter (PM_2.5_) concentrations. PATS+ contains a photometer that responds to an average concentration of particles and measures a very wide range of particle concentrations with a lower and upper particulate matter detection limit of 10–20 μg/m^3^ and 30,000 to 50,000 μg/m^3^, respectively. Both initial and end zero-ing was applied before and after each sample measurement using an air-tight zero-ing box with a high efficiency particulate absorbing (HEPA) filter according to the manufacturer’s instructions, while one-minute logging intervals were used to measure PM_2.5_ concentrations during 24 h. The PATS+ photoelectric signals respond consistently and predictably to increasing concentrations of particulate matter, thus, are feasible and affordable for use in rural settings of low-income countries [25].

The particle monitoring instruments were positioned in the cooking area in accordance with the standard placement protocols (criteria are specified in the next section). Since cooking for the family is usually the responsibility of the mother, the data collector asked for the mother’s plan for the next 24 h before starting the process of installing the instrument. Households who had plans to travel or attend any festivity or local holiday celebration in their neighbourhood were given another appointment. This is because the cooking processes may not take place and/or unusual cooking practices may occur in their neighborhood thus increasing the particulate matter concentration in their house originating in the ambient environment. Fridays were specifically excluded because the market held in the city on these days is attended by nearly all rural mothers. The measurement starting time varied for practical reasons, but 24-h measures were obtained from all the households. Peaks in PM_2.5_ concentrations were checked against the cooking time of the meals during the data collection period (i.e., cooking activities in the last 24 h were recorded immediately after the measurement by asking the mother).

### 2.5. Particulate Matter Data Quality Assurance

The PM_2.5_ data was collected during two seasons to generate representative measurements. The fourth author (MA), performed the data collection in close collaboration with the first author (MT). The data collector and field guides explained the procedures to the mother and the male household heads. They kept a watch on the instruments to avoid loss of data due to a malfunctions in the field. The following criteria were considered to ensure the validity of the measurements: a height of 1.5 metres relating to the approximate breathing zone of a standing woman, unobstructed airflow, location away from doors and windows to avoid measuring ambient air entering the room, placement at least one metre away from the edge of an active cooking stove to avoid undue influence of point source and placement in a safe and dry place. The instruments (*n* = 3) were zeroed in a zero-ing box for 30 min before and after deployment in their respective households. We did not compare our findings to gravimetric measurements in this study. A comparison was made between PM_2.5_ concentrations determined using PATS+ and gravimetric measurements in kitchens of families using traditional wood-burning cooking stoves in Guatemala. The resulting strong linear correlation (R^2^ > 0.90) [25] indicated that the PATS+ photoelectric signals correlated well with the gravimetric measurements across the range of particulate matter concentrations under real-world conditions and that the instrument is sufficiently accurate.

### 2.6. Statistical Analysis

The following descriptive statistics were generated: (a) frequency and percentage for categorical variables and (b) independent sampling t-tests to compare means of two different groups and checking for significant differences between these means using IBM SPSS Statistics for Windows, Version 24.0 (IBM Corp., Armonk, NY, USA). When checked prior to t-test analysis, the data had a tolerable level of skewness (1.1) and the Levene test did not indicate inequality in the variances or *p*-values above our selected alpha threshold of 0.05. Correlation analysis was used to assess existence of possible linear association or statistical relationships between the 24-h mean concentrations of PM_2.5_ and reported cooking duration over the same period.

### 2.7. Ethical Approval

Ethical approval was obtained from the Institutional Review Board of the College of Health Sciences of Addis Ababa University, Ethiopia (004/16/SPH). Permission was obtained to conduct the research and for the installation of the measuring instrument both from the mother and the male household head. All interviews were conducted after obtaining informed consent, while anonymity and confidentiality were maintained throughout the study.

## 3. Results

Data from 147 households in five villages were included in the analysis. Three of the 150 households selected were excluded. In one of these three households no cooking occurred due to unexpected travel of the mother while the other two did not follow their normal cooking routines. The majority (86%) of the houses were tukul houses (a traditional circular hut with thatched conical roof) and one third of them had a family size of six and above. Wood was the only primary fuel used while crop residues alone or together with animal dung accounted for over 90% of the secondary fuel used during the dry season or when available. Around 70% of the households used traditional three-clay stoves. The 24-h mean and median PM_2.5_ concentration was 410 and 340 µg/m^3^, respectively (Table 1).

**Figure 1:**
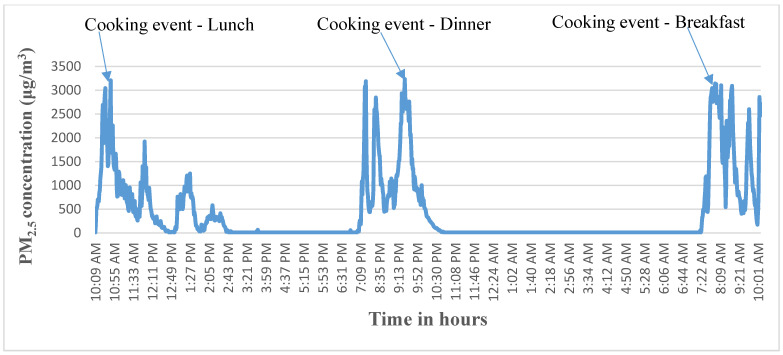
Results from a representative household measurement of PM_2.5_ during 24 h, including cooking events as reported by the mother and peak PM_2.5_ concentrations as determined by a Particle and Temperature Sensor (PATS+) instrument positioned in the cooking area.

Figure 1 shows the peak concentrations of PM_2.5_ with 24-h mean 420 µg/m^3^ on a representative day from one household in the Wurib village. As shown in the Figure, the peak concentrations occurred during the usual cooking and meal times in the household (lunch, dinner and breakfast, respectively) and also matched the activity report provided by the mother of the household. There are two or more peaks in one cooking duration indicating the cooking of bread and stew and the preparation of coffee after the meal.

### 3.1. Mean PM_2.5_ Concentration in Relation to Cooking and Housing Conditions

The mean PM_2.5_ concentration in the households was very high at 410 μg/m^3^ ranging from 100 to 1200 μg/m^3^. These mean PM_2.5_ concentrations showed statistically significant differences by stove type, having the door open during cooking at night and having a window open during cooking (Table 2). Households using only traditional stoves and those not keeping the door or a window open had significantly higher mean concentration compared to their counterparts.

### 3.2. Correlation between 24-h Mean PM_2.5_ and Total Duration of Cooking

**Figure 2:**
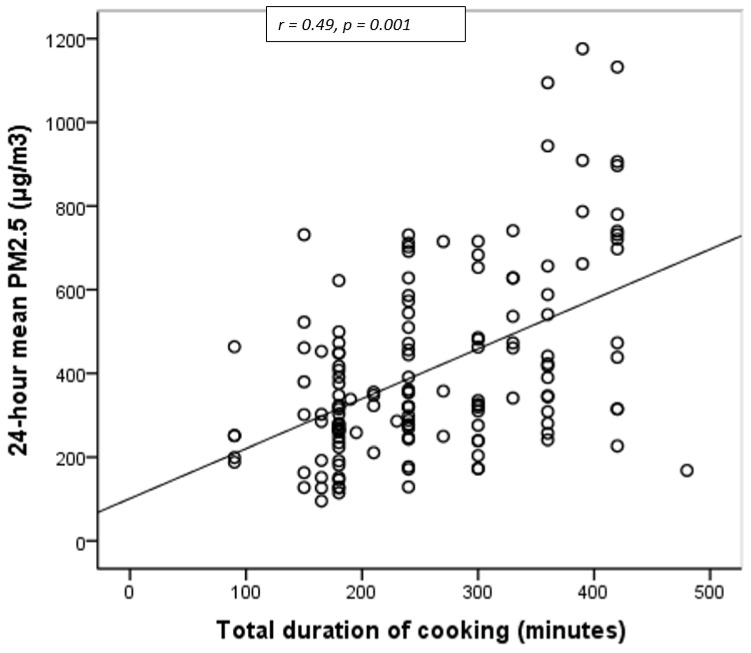
Correlation between 24-h mean PM_2.5_ (μg/m^3^) and total duration of cooking (minutes) determined in 147 households in rural Butajira, Ethiopia.

The 24-h mean measure of PM_2.5_ (μg/m^3^) and self-reported cooking times showed a positive correlation coefficient of r = 0.49 and a *p*-value 0.001. This established that longer cooking times generated more particulate matter (Figure 2).

## 4. Discussion

All households in this study had very high levels of household air pollution, higher than previously measured in Uganda and Ethiopia [6] and other low-income countries [7,26,27,28,29]. The 24-h mean concentration level of PM_2.5_ was 410 μg/m^3^ ranging from 100 to 1200 μg/m^3^, which is 16 times higher than the limit of 25 μg/m^3^ recommended by the WHO 24-h mean air quality guideline. Even the lowest 24-h mean concentration was four times higher than the guideline limit set by the WHO. Concentrations in the cooking area similar to that reported in this study has been reported from India [26]. Other studies from Ethiopia [30]; Ghana [31] and India [32] have also showed high 24-h mean particulate matter concentrations, comparable with the results from our study. However, it should be noted that the vast majority of the households in the present study take the form of one-room dwellings with the cooking area located in the middle and the sleeping area located directly behind it. Thus, the cooking is carried out directly beside the living space. The PM_2.5_ concentration in the dwellings in Ethiopia was also considerably higher compared with the mean value (160 μg/m^3^) reported for the living/cooking dwellings in the Indian study [32]. Other possible reasons for differences in the mean PM_2.5_ concentrations include differences in the measuring instruments, the amount and frequency of cooking, the type of stoves and houses, poor ventilation due to inadequate windows/any other openings and other cooking procedures.

The high 24-h mean PM_2.5_ concentration in the current study indicates that there has been no or little improvement in housing conditions and fuel use in the locality over nearly two decades [33]. A study of indoor air pollution from households in the same area between the years 2000 and 2002 found high levels of NO_2_, another indicator of indoor air pollution. The current fuel types, wood, crop residues and animal dung, were also the common fuel types used at that time [33]. There has been a lack of political priority to provide cleaner energy sources to rural communities. The recent industrial developments have been monopolising the limited electric power supplies available during the country’s rapid economic growth. It is also worth considering the problem of household air pollution and impact of this on the health of the population as referred to in the discussion of the Grand Ethiopian Renaissance Dam (GERD) which is supposed to relieve Ethiopians’ acute energy shortage [34]. Wood burning has not only consequences which impact on health but also a significant negative impact on the environment and economic growth of Sub-Saharan countries [33,35] and possibly contribute to ambient air pollution [36,37,38].

The 24-h mean PM_2.5_ concentration did not show a statistically significant difference between the wet and the dry seasons in contrast to previous studies reporting the concentration to be higher during wet/rainy seasons [39,40]. This could be linked to the use of crop residues or animal dung as a fuel source during the dry, post-harvesting season in our study area, which might result in a higher pollution level due to the combustion process which is inefficient compared with wood. Both the current finding and our previous qualitative study in the same area indicated wood as the primary fuel source during the seasons when crop residues or animal dung were not available [41].

The use of traditional three-clay cooking stoves resulted in significantly higher concentrations of particulate emissions compared with locally produced improved cookstoves. The more complete burning process and combustion using improved cookstoves has the potential to reduce pollution levels. Evidence from previous studies in different parts of the world, and Ethiopia, indicate that locally available improved cookstoves had lower emissions of particulate matter and reduced household air pollution [42,43,44,45,46,47]. This was also supported by the participants of our previous qualitative research from the same community who witnessed that cooking using improved cookstoves reduced the problem of wood smoke [41]. Some locally available improved cookstoves have been tested and found to reduce emission of particulate matter by 46% during traditional cooking in the dwelling [48]. However, other studies did not find statistically significant differences in the concentration of the particulate matter by the type of stove used in field studies with the exception of modest improvements in emission levels [49,50]. Use of improved cookstoves alone may not effectively reduce the health impacts unless supported by behavioural interventions as evidenced by a randomized controlled trial in Malawi [51].

Ventilating the cooking area by having the door or any windows open during the active cooking period showed a statistically significant reduction of the 24-h mean concentration of PM_2.5_. Previous studies from different countries reported similar findings of lower pollution concentrations in houses with adequate ventilation practices and availability of more doors and windows [52,53,54]. Optimizing ventilation has been considered as an important practice for reducing the health risks [14]. The practice of not opening a window—or if there are no windows—is due to the cultural perception related to security or a fear of theft in the area as reported in our previous study [41] and might also be related with the weather conditions [54].

In the current study, the 24-h mean PM_2.5_ concentration was positively correlated to the cooking time; the longer the cooking process, the higher emission of particulate matter. This is consistent with the findings of a study from India [32]. In contrast, there has been a study from the same region reporting no difference in the level of PM_2.5_ concentration based on the time spent on cooking [30]. This difference might be derived from the type of statistical analysis applied. We have recorded time as a continuous variable rather than a discrete variable as done in that study.

We did not find any statistically significant difference in the 24-h mean PM_2.5_ concentration by family size, type of house, frequency of cooking or season during the measurement. However, it is important to note that the particulate matter concentration in all the cases was excessively high. As long as solid fuel and open cooking is used, the health risks, due to the exposure of PM_2.5_, are inevitable in the community.

A strength of this study was that it was conducted in both dry and wet seasons; thus, the problem of household air pollution is a public year-round health issue. A limitation of the study was not using gravimetric analysis of particulate matter since only optical measurements were used. However, we followed the protocol and standard operation procedure to ensure accurate measurement. In addition, a laboratory test of the equipment to normalize and calibrate their response was done with wood smoke and it has been used for field measurements by other research groups worldwide [25]. Another limitation was that the 24-h sampling period per household was conducted only once, thus we were not able to see variation at the same household level.

## 5. Conclusions and Recommendation

The 24-h mean PM_2.5_ concentration was up to 16 times higher than the WHO 24-h recommended guideline limit. We thus concluded that particularly mothers and children, who spend the most time at the domestic hearth are at risk of the adverse health effects of solid fuel combustion in rural Ethiopia. We thus propose that effective short- and long-term interventions are needed to protect the health of the population in the country, given the large rural population. We recommend a follow-up study to further evaluate the magnitude of health effects related to the high exposure to household air pollution in the locality. We also recommend the implementation of suitable and sustainable strategies to reduce exposure.

## Figures and Tables

**Table 1:** Background characteristics and measurements of household particulate matter (PM_2.5_).

Characteristics		Households, *n* (%)
Villages	Dirama	25 (17)
	Dobena	30 (20)
	Misrak Meskan	29 (20)
	Shersherebedo	26 (18)
	Wurib	37 (25)
Type of house	Tukul	126 (86)
	Tin	21 (14)
Family size	Five or less	54 (37)
	Six and above	93 (63)
Season (during data collection)	Rainy (Wet)	65 (44)
	Dry	82 (56)
Frequency of cooking in 24 h	Three times or less	65 (44)
	Four times	82 (56)
Primary fuel type	Wood	147 (100)
Type of stove	Traditional three-clay stove	100 (68)
	Improved/moveable	47 (32)
Secondary fuel type	Crop residue only	72 (49)
	Dung only	2 (1)
	Crop residue and dung	66 (45)
	Charcoal	7 (5)
Duration (minutes) of cooking in 24 h *	Mean (95% CI)	258 (244–273)
PM_2.5_ (μg/m^3^)	Mean (95% CI)	410 (370–450)
	Median (Min-Max)	340 (100–1200)
	Geometric mean	360
	25th percentile	260
	75th percentile	510
	95th percentile	850

* Includes coffee preparation.

**Table 2:** Mean PM_2.5_ concentration in relation to cooking and housing conditions.

Characteristics (*n*)		Mean PM_2.5_ in μg/m^3^ (SD)	95% CI	*p*-Value
Family size	Five or less (85)	390 (210)	340–430	0.208
	Six or above (62)	440 (230)	380–500	
Type of house	Tukul house (126)	420 (220)	380–430	0.092
	Tin house (21)	330 (210)	240–430	
Type of stove	Traditional three-clay (100)	440 (240)	390–490	0.009 *
	Improved/moveable (47)	340 (140)	300–380	
Door opened during cooking at night	No (52)	470 (240)	400–530	0.017 *
	Yes (95)	380 (200)	340–530	
Window open during cooking	No (102)	430 (230)	390–480	0.032 *
	Yes (45)	350 (200)	300–410	
Cooking frequency	Three or less (110)	420 (230)	370–460	0.534
	Four times (37)	390 (193)	330–450	
Season of the year	Rainy/Wet (65)	420 (240)	360–480	0.542
	Dry (82)	400 (210)	350–440	

SD = Standard deviation * significant at significance level of 0.05.

## Data Availability

The original raw data used in this study is available from the corresponding author and can be presented upon reasonable request.

## References

[1] World Health Organization Household Air Pollution and Health. http://www.who.int/mediacentre/factsheets/fs292/en/.

[2] Champion W.M., Connors L., Montoya L.D. (2017). Emission factors of fine particulate matter, organic and elemental carbon, carbon monoxide, and carbon dioxide for four solid fuels commonly used in residential heating by the U.S. Navajo Nation. J. Air Waste Manag. Assoc..

[3] Du Y., Xu X., Chu M., Guo Y., Wang J. (2016). Air particulate matter and cardiovascular disease: The epidemiological, biomedical and clinical evidence. J. Thorac. Dis..

[4] Smith K.R., McCracken J.P., Weber M.W., Hubbard A., Jenny A., Thompson L.M., Balmes J., Diaz A., Arana B., Bruce N. (2011). Effect of reduction in household air pollution on childhood pneumonia in Guatemala (RESPIRE): A randomised controlled trial. Lancet.

[5] Sharma D., Jain S. (2019). Impact of intervention of biomass cookstove technologies and kitchen characteristics on indoor air quality and human exposure in rural settings of India. Environ. Int..

[6] Okello G., Devereux G., Semple S. (2018). Women and girls in resource poor countries experience much greater exposure to household air pollutants than men: Results from Uganda and Ethiopia. Environ. Int..

[7] Pramitha E., Haryanto B. (2019). Effect of Exposure to 2.5 μm Indoor Particulate Matter on Adult Lung Function in Jakarta. Osong Public Health Res. Perspect..

[8] Gordon S., Bruce N.G., Grigg J., Hibberd P.L., Kurmi O.P., Lam K.B.H., Mortimer K., Asante K.P., Balakrishnan K., Balmes J. (2014). Respiratory risks from household air pollution in low and middle income countries. Lancet Respir. Med..

[9] Cao Q., Rui G., Liang Y. (2018). Study on PM2.5 pollution and the mortality due to lung cancer in China based on geographic weighted regression model. BMC Public Health.

[10] Pokhrel A.K., Smith K.R., Khalakdina A., Deuja A., Bates M.N. (2005). Case–control study of indoor cooking smoke exposure and cataract in Nepal and India. Int. J. Epidemiol..

[11] Dherani M., Pope D., Mascarenhas M., Smith K.R., Weber M., Bruce N. (2008). Indoor air pollution from unprocessed solid fuel use and pneumonia risk in children aged under five years: A systematic review and meta-analysis. Bull. World Health Organ..

[12] Bede-Ojimadu O., Orisakwe O.E. (2020). Exposure to Wood Smoke and Associated Health Effects in Sub-Saharan Africa: A Systematic Review. Ann. Glob. Health.

[13] World Health Organization (2006). WHO Air Quality Guidelines for Particulate Matter, Ozone, Nitrogen Dioxide and Sulfur Dioxide: Global Update Summary of Risk Assessment.

[14] Asikainen A., Carrer P., Kephalopoulos S., Fernandes E.D.O., Wargocki P., Hänninen O. (2016). Reducing burden of disease from residential indoor air exposures in Europe (HEALTHVENT project). Environ. Health.

[15] Desai M., Mehta S., Smith K. (2004). Indoor Smoke from Solid Fuels: Assessing the Environmental Burden of Disease at National and Local Levels.

[16] UN (2015). The World Population Prospects: 2015 Revision.

[17] Aboubacar B., Deyi X., Razak M.Y.A., Leyla B.H. (2018). The Effect of PM2.5 from Household Combustion on Life Expectancy in Sub-Saharan Africa. Int. J. Environ. Res. Public Health.

[18] Owili P.O., Muga M.A., Pan W.-C., Kuo H.-W. (2017). Cooking fuel and risk of under-five mortality in 23 Sub-Saharan African countries: A population-based study. Int. J. Environ. Health Res..

[19] Tamire M., Addissie A., Kumie A., Husmark E., Skovbjerg S., Andersson R., Lärstad M. (2019). Respiratory Symptoms and Lung Function among Ethiopian Women in Relation to Household Fuel Use. Int. J. Environ. Res. Public Health.

[20] Demelash H., Motbainor A., Nigatu D., Gashaw K., Melese A. (2015). Risk factors for low birth weight in Bale zone hospitals, South-East Ethiopia: A case–control study. BMC Pregnancy Childbirth.

[21] Biruck Y., Suleiman H., Asfaw A. (2011). Household fuel use and acute respiratory infections among younger children: An exposure assessment in Shebedino Wereda, Southern Ethiopia. Afr. J. Health Sci..

[22] Admasie A., Kumie A., Worku A. (2018). Children under Five from Houses of Unclean Fuel Sources and Poorly Ventilated Houses Have Higher Odds of Suffering from Acute Respiratory Infection in Wolaita-Sodo, Southern Ethiopia: A Case-Control Study. J. Environ. Public Health.

[23] Gebremariam T.H., Binegdie A.B., Mitiku A.S., Ashagrie A.W., Gebrehiwot K.G., Huluka D.K., Sherman C.B., Schluger N.W. (2017). Level of asthma control and risk factors for poor asthma control among clinic patients seen at a Referral Hospital in Addis Ababa, Ethiopia. BMC Res. Notes.

[24] Shamebo D., Sandström A., Wall S. (1992). The Butajira Rural Health Project in Ethiopia: Epidemiological Surveillance for Research and Intervention in Primary Health Care. Scand. J. Prim. Health Care.

[25] Pillarisetti A., Allen T., Ruiz-Mercado I., Edwards R., Chowdhury Z., Garland C., Hill L.D., Johnson M., Litton C.D., Lam N.L. (2017). Small, Smart, Fast, and Cheap: Microchip-Based Sensors to Estimate Air Pollution Exposures in Rural Households. Sensors.

[26] Mukhopadhyay R., Sambandam S., Pillarisetti A., Jack D., Mukhopadhyay K., Balakrishnan K., Vaswani M., Bates M.N., Kinney P.L., Arora N. (2012). Cooking practices, air quality, and the acceptability of advanced cookstoves in Haryana, India: An exploratory study to inform large-scale interventions. Glob. Health Action.

[27] Nasar Z.A., Colbeck I., Ali Z., Ahmad S. (2013). Indoor particulate matter in developing countries: A case study in Pakistan and potential intervention strategies. Environ. Res. Lett..

[28] Dionisio K.L., Howie S.R.C., Dominici F., Fornace K., Spengler J.D., Adegbola R.A., Ezzati M. (2012). Household Concentrations and Exposure of Children to Particulate Matter from Biomass Fuels in The Gambia. Environ. Sci. Technol..

[29] McCracken J.P., Schwartz J., Díaz A., Bruce N., Smith K.R. (2013). Longitudinal Relationship between Personal CO and Personal PM2.5 among Women Cooking with Woodfired Cookstoves in Guatemala. PLoS ONE.

[30] Admasie A., Kumie A., Worku A., Tsehayu W. (2019). Household fine particulate matter (PM2.5) concentrations from cooking fuels: The case in an urban setting, Wolaita Sodo, Ethiopia. Air Qual. Atmos. Health.

[31] Van Vliet E.D., Asante K., Jack D.W., Kinney P.L., Whyatt R.M., Chillrud S.N., Abokyi L., Zandoh C., Owusu-Agyei S. (2013). Personal exposures to fine particulate matter and black carbon in households cooking with biomass fuels in rural Ghana. Environ. Res..

[32] Balakrishnan K., Ghosh S., Ganguli B., Sambandam S., Bruce N., Barnes D.F., Smith K.R. (2013). State and national household concentrations of PM2.5 from solid cookfuel use: Results from measurements and modeling in India for estimation of the global burden of disease. Environ. Health.

[33] Kumie A., Emmelin A., Wahlberg S., Berhane Y., Ali A., Mekonnen E., Brandstrom D. (2009). Magnitude of indoor NO2from biomass fuels in rural settings of Ethiopia. Indoor Air.

[34] Teferi Taye M., Tadesse T., Senay G., Block P. (2016). The Grand Ethiopian Renaissance Dam: Source of cooperation or contention?. J. Water Resour. Plan. Manag..

[35] Sulaiman C., Abdul-Rahim A. (2020). The Impact of Wood Fuel Energy on Economic Growth in Sub-Saharan Africa: Dynamic Macro-Panel Approach. Sustainability.

[36] Johnston H.J., Mueller W., Steinle S., Vardoulakis S., Tantrakarnapa K., Loh M., Cherrie J.W. (2019). How Harmful Is Particulate Matter Emitted from Biomass Burning? A Thailand Perspective. Curr. Pollut. Rep..

[37] Desservettaz M., Phillips F., Naylor T., Price O., Samson S., Kirkwood J., Paton-Walsh C. (2019). Air Quality Impacts of Smoke from Hazard Reduction Burns and Domestic Wood Heating in Western Sydney. Atmosphere.

[38] Zhang S., Yuval B.D., Raz R. (2020). Predictors of the Indoor-to-Outdoor Ratio of Particle Number Concentrations in Israel. Atmosphere.

[39] Li S., Xu J., Jiang Z., Luo Y., Yang Y., Yu J. (2019). Correlation between indoor air pollution and adult respiratory health in Zunyi City in Southwest China: Situation in two different seasons. BMC Public Health.

[40] Gurley E.S., Salje H., Homaira N., Ram P.K., Haque R., Petri W.A., Bresee J., Moss W.J., Luby S.P., Breysse P. (2013). Seasonal concentrations and determinants of indoor particulate matter in a low-income community in Dhaka, Bangladesh. Environ. Res..

[41] Tamire M., Addissie A., Skovbjerg S., Andersson R., Lärstad M. (2018). Socio-Cultural Reasons and Community Perceptions Regarding Indoor Cooking Using Biomass Fuel and Traditional Stoves in Rural Ethiopia: A Qualitative Study. Int. J. Environ. Res. Public Health.

[42] Pilishvili T., Loo J.D., Schrag S., Stanistreet D., Christensen B., Yip F., Nyagol R., Quick R., Sage M., Bruce N. (2016). Effectiveness of Six Improved Cookstoves in Reducing Household Air Pollution and Their Acceptability in Rural Western Kenya. PLoS ONE.

[43] Begum B. (2015). Comparison of a Traditional Cook Stove with Improved Cook Stoves Based on Their Emission Characteristics. Nucl. Sci. Appl..

[44] Mamuye F., Lemma B., Woldeamanuel T. (2018). Emissions and fuel use performance of two improved stoves and determinants of their adoption in Dodola, southeastern Ethiopia. Sustain. Environ. Res..

[45] Yip F., Christensen B., Sircar K., Naeher L., Bruce N., Pennise D., Lozier M., Pilishvili T., Farrar J.L., Stanistreet D. (2017). Assessment of traditional and improved stove use on household air pollution and personal exposures in rural western Kenya. Environ. Int..

[46] Embiale A., Chandravanshi B.S., Zewge F., Sahle-Demessie E. (2021). Indoor air pollution from cook-stoves during Injera baking in Ethiopia, exposure, and health risk assessment. Arch. Environ. Occup. Health.

[47] Thomas E., Wickramasinghe K., Mendis S., Roberts N., Foster C. (2015). Improved stove interventions to reduce household air pollution in low and middle income countries: A descriptive systematic review Environmental health. BMC Public Health.

[48] Adane M.M., Alene G.D., Mereta S.T. (2021). Biomass-fuelled improved cookstove intervention to prevent household air pollution in Northwest Ethiopia: A cluster randomized controlled trial. Environ. Health Prev. Med..

[49] Balakrishnan K., Sambandam S., Ghosh S., Mukhopadhyay K., Vaswani M., Arora N.K., Jack D., Pillariseti A., Bates M.N., Smith K.R. (2015). Household Air Pollution Exposures of Pregnant Women Receiving Advanced Combustion Cookstoves in India: Implications for Intervention. Ann. Glob. Health.

[50] Sanbata H., Asfaw A., Kumie A. (2014). Indoor air pollution in slum neighbourhoods of Addis Ababa, Ethiopia. Atmos. Environ..

[51] Mortimer K., Ndamala C.B., Naunje A.W., Malava J., Katundu C., Weston W., Havens D., Pope D., Bruce N.G., Nyirenda M. (2017). A cleaner burning biomass-fuelled cookstove intervention to prevent pneumonia in children under 5 years old in rural Malawi (the Cooking and Pneumonia Study): A cluster randomised controlled trial. Lancet.

[52] Majdan M., Svaro M., Muendo R., Taylor M., Kralova Z. (2015). Effectiveness of various ventilation systems in reducing exposure to biomass related particles: A real-life experiment. Ann. Trop. Med. Public Health.

[53] Parajuli I., Lee H., Shrestha K.R. (2016). Indoor Air Quality and ventilation assessment of rural mountainous households of Nepal. Int. J. Sustain. Built Environ..

[54] Weaver A.M., Sharmin I., Parveen S., Crabtree-Ide C., Luby S.P., Mu L., Goswami D., Rudra C., Ram P.K., Fry A.M. (2017). Pilot Intervention Study of Household Ventilation and Fine Particulate Matter Concentrations in a Low-Income Urban Area, Dhaka, Bangladesh. Am. J. Trop. Med. Hyg..

